# Sleep in 22q11.2 Deletion Syndrome: Current Findings, Challenges, and Future Directions

**DOI:** 10.1007/s11920-023-01444-6

**Published:** 2023-09-18

**Authors:** Kathleen P. O’Hora, Charles H. Schleifer, Carrie E. Bearden

**Affiliations:** 1 Department of Psychiatry and Biobehavioral Sciences, Semel Institute for Neuroscience and Human Behavior, University of California, 760 Westwood Plaza, Los Angeles, CA 90095 USA; 2 Neuroscience Interdepartmental Program, University of California, Los Angeles, CA USA; 3 David Geffen School of Medicine, University of California, Los Angeles, CA USA; 4 Department of Psychology, University of California, Los Angeles, CA USA

**Keywords:** 22q11.2, Velocardiofacial Syndrome, DiGeorge Syndrome, Sleep, Psychotic spectrum disorders, Autism, Copy number variation, Sleep spindles

## Abstract

**Purpose of Review:**

To summarize current literature available on sleep in 22q11.2 Deletion Syndrome (22q11.2DS; Velocardiofacial or DiGeorge Syndrome), a neurogenetic disorder caused by a hemizygous deletion in a genomic region critical for neurodevelopment. Due to the greatly increased risk of developmental psychiatric disorders (e.g., autism and schizophrenia) in 22q11.2DS, this review focuses on clinical correlates of sleep disturbances and potential neurobiological underpinnings of these relationships.

**Recent Findings:**

Sleep disturbances are widely prevalent in 22q11.2DS and are associated with worse behavioral, psychiatric, and physical health outcomes. There are reports of sleep architecture and sleep neurophysiology differences, but the literature is limited by logistical challenges posed by objective sleep measures, resulting in small study samples to date.

**Summary:**

Sleep disturbances in 22q11.2DS are prevalent and have a substantial impact on well-being. Further investigation of sleep in 22q11.2DS utilizing multimodal sleep assessments has the potential to provide new insight into neurobiological mechanisms and a potential trans-diagnostic treatment target in 22q11.2DS.

## Introduction

The 22q11.2 locus is a genomic hotspot with highly conserved genes critical to neurodevelopment [1]. 22q11.2 Deletion Syndrome (22q11.2DS; also known as DiGeorge Syndrome) results from a ~ 1.5 to 2.6 Megabase hemizygous deletion in this locus (~ 46 protein-coding genes) and is one of the most common genetic syndromes, occurring in approximately 1 in 4000 live births [2]. 22q11.2DS is associated with developmental delay (DD) and intellectual disability (ID), as well as high rates of autism spectrum disorder (ASD) and attention-deficit hyperactivity disorder (ADHD) [3]. Additionally, 22q11.2DS is one of the greatest known genetic risk factors for psychosis spectrum disorders (PSD), including schizophrenia [4]. Congenital malformations, including cardiac and craniofacial abnormalities, are also common in 22q11.2DS. Due to the well-documented large effects on neurodevelopmental behavioral phenotypes, 22q11.2DS serves as a compelling model for investigating genetic, brain, and behavior relationships relevant to neurodevelopmental psychiatric disorders (NPDs). See Table 1 for a glossary of acronyms.

**Table 1:** Glossary of abbreviations

**Acronym**	**Definition**
22q11.2DS	22q11.2 Deletion Syndrome
ADHD	Attention-deficit hyperactivity disorder
AHI	Apnea–hypopnea index
ASD	Autism spectrum disorder
CBT-I	Cognitive behavioral therapy for insomnia
CHR	Clinical high risk for psychosis
CSHQ	Children Sleep Habits Questionnaire
DD	Developmental delay
EEG	Electroencephalography
EKG	Electrocardiogram
EMG	Electromyogram
EOG	Electrooculogram
GI	Gastrointestinal
ID	Intellectual disability
IL-10	Interleukin 10
IL-6	Interleukin 6
NPD	Neurodevelopmental psychiatric disorder
NREM	Non-rapid eye movement
OSA	Obstructive sleep apnea
PSD	Psychotic spectrum disorders
PSG	Polysomnography
PSQI	Pittsburgh Sleep Quality Index
RBD	Rapid Eye Movement behavior disorder
REM	Rapid eye movement
rs-fMRI	Resting-state functional magnetic resonance imaging
SDB	Sleep disordered breathing
SE	Sleep efficiency
SOL	Sleep onset latency
SW	Slow wave
TD	Typically developing
TIB	Time in bed
TST	Total sleep time
WASO	Wake after sleep onset

Sleep disturbance is a common, core feature of many NPDs associated with 22q11.2DS [5–10]. There is a bidirectional relationship between sleep and psychiatric symptoms, by which worse sleep can worsen psychiatric symptoms [11], while worsening psychiatric symptoms may also cause sleep disturbances. This bidirectional relationship suggests that sleep improvement can improve psychiatric well-being [12]. Further, sleep is an evolutionarily conserved process that provides insight into underlying neurobiology, providing rich opportunity for practical clinical translation. Given this, studying sleep in individuals with NPDs has the potential to unlock novel trans-diagnostic biomarkers, etiological insight, and intervention targets for behaviors and symptoms impacting quality of life.

Despite the close relationship between sleep disturbances and NPDs, the literature on sleep in 22q11.2DS is scarce. The majority of existing studies rely on subjectively reported measures of sleep. These subjective measures are a mix of parent-report questionnaires (e.g., Child Sleep Habits Questionnaire [13] (CSHQ)), self-report questionnaires (i.e., Pittsburgh Sleep Quality Index [14] (PSQI)), and/or clinician ratings from sleep modules on clinical interviews, which are based on participant or parent report (e.g., Structured Interview for Psychosis Risk Syndromes [15] and Child and Adolescent Psychiatric Assessment [16]). A few studies have utilized objective measures of sleep, including wrist actigraphy and overnight polysomnography (PSG) or high-density electroencephalography (EEG). Wrist actigraphy is a method of distinguishing sleep from wake using physical activity measured by an accelerometer worn on the wrist [17, 18]. Actigraphy is the basis of most consumer wearable sleep monitoring devices and provides information on sleep continuity and timing, such as total sleep time (TST), wake after sleep onset (WASO), number of night awakenings, and sleep/rise time. While actigraphy is a well-validated method of estimating sleep, it is limited because it is not a direct measure of sleep and cannot reliably measure underlying sleep architecture (i.e., sleep staging) or biological processes regulating sleep. Therefore, methods using EEG are considered the gold standard for measuring sleep neurophysiology. Some studies choose to employ clinical PSG, which includes peripheral sensors such as a pulse oximeter, plethysmography belts, electrocardiogram (EKG), and a nasal cannula, in addition to scalp EEG, electrooculogram (EOG), and electromyogram (EMG) sensors, to screen for sleep disorders (i.e., sleep-disordered breathing (SDB)). However, other studies opt for a higher density montage of scalp EEG in place of peripheral sensors to characterize the topology of sleep neurophysiology. Each class of sleep measure provides information about different aspects of sleep. For example, sleep questionnaires provide information on subjective sleep experience, which may differ from what is observed on objectively measured sleep. Each tool has advantages and limitations, depending on the research questions, that should be considered when interpreting results.

In this comprehensive review, we will summarize the literature on sleep in 22q11.2DS, outline methodological challenges, and propose future directions to move the field forward to fully realize the full translational potential of sleep research in 22q11.2DS.

## Behavioral Sleep Phenotype

Studies including subjectively reported sleep measures have found that individuals with 22q11.2DS, including young children [19], experience increased general sleep disturbances and lower sleep quality [19–21, 22•, 23•, 24, 25•, 26, 27•, 28••, 29•]. Subjects with 22q11.2DS reported worse overall sleep compared to typically developing (TD) controls in studies that included a direct comparison between 22q11.2DS and TD controls [19, 20, 22•, 23•, 28••, 29•]. One study categorized 30% of 22q11.2DS participants as overall poor sleepers, compared to 2% of control subjects, based on multiple self/parent report and clinician-rated measures [20]. In studies without a comparison group, 22q11.2DS subjects endorse high levels of sleep disturbance relative to the clinical scoring criteria of each respective instrument [21, 24, 26]. In subjective assessments that assessed specific aspects of sleep disturbance, 22qDel carriers exhibited increased bedtime resistance, sleep anxiety, night waking, and sleepiness [21].

### Sleep Continuity

Individuals with 22q11.2DS exhibit more problems initiating and maintaining sleep, including longer SOL [21, 22•, 25•] and increased WASO [25•] based on subjectively reported sleep measures, regardless of reporter. However, three studies utilizing objective sleep measures (actigraphy [29•] and PSG [27•, 28••]) found no differences in measures of sleep maintenance in 22q11.2DS compared to TD controls [27•, 28••, 29•], and only one found evidence of difficulty initiating sleep [27•]. Actigraphy measures in one study identified increases in TST and time in bed (TIB) [29•], which were replicated by one of the two EEG sleep studies in 22q11.2DS [27•, 28••]. A summary of methods and key findings of studies examining sleep disturbances in 22q11.2DS is provided in Table 2.

**Table 2:** Summary of literature on sleep in 22q11.2 Deletion Syndrome

**Author**	**Year**	**Type**	**Sleep measures**	**Sample**	**Ages**	**Key findings**
• Yirmiya	2020	Human: self-report	Pittsburgh Sleep Quality Index (PSQI)	22q11.2DS = 33 TD = 24	8–60	• ↓ Sleep quality in 22q11.2DS relative to controls • ↑ Sleep disturbance associated with ↓ cognition • ↑ Sleep disturbance associated with ↑ IL-6
Arganbright	2020	Human: parent report	Children’s Sleep Habits Questionnaire (CSHQ)	22q11.2DS = 30	1–15	• Sleep problems in 29/30 children • Younger age related to ↑ bedtime resistance, night wakings and sleep-disordered breathing, and ↓ sleep onset delay
• Moulding	2020	Human: clinical interview	Child and Psychiatric Assessment (CAPA)	22q11.2DS = 140 TD = 65	6–17	• ↑ Insomnia and restless sleep in 22q11.2DS • Restless sleep related to ↑ ADHD and anxiety, and ↓ executive function • Insomnia related to ↑ anxiety, conduct disorder and developmental coordination disorder
Leader	2020	Human: parent report	CSHQ	22q11.2DS = 149	3–18	• Sleep problems in 91% of sample • Sleep problems associated with ↑ gastrointestinal symptoms
• Hyde	2021	Human: self-report	Daily sleep diary	22q11.2DS = 31 TD = 24	18–49	• ↑ SOL and WASO in 22q11.2DS but no significant group difference in subjective sleep quality • Stronger relationship between sleep and affect in 22q11.2DS
O'Hora	2022	Human: self/parent report	Sleep-related items from clinical interview and parent-report	22q11.2DS = 107 22qDup = 42 TD = 88	6–45	• ↑ General sleep disturbance in 22q11.2DS • ↑ General sleep disturbance associated with ↑psychosis-risk symptoms, ↑ ASD symptomatology, ↑somatic complaints, ↑anxiety/depression, ↑ thought problems, ↑aggressive behaviors, and ↓ real-world executive function
••Donnelly	2022	Human: EEG	High-density ambulatory PSG	22q11.2DS = 28 TD = 17	6–20	• ↑ N3 sleep and ↓ N1 and REM in 22q11.2DS • ↑ Slow-wave amplitude; ↑ spindle amplitude, frequency, density, and slow-wave coupling in 22q11.2DS
• Mauro	2022	Human: EEG and self-report	• PSQI • REM behavior disorder (RBD) questionnaire • Video PSG	22q11.2DS = 26	18–51	• High RBD score in 7/26 patients • Poor subjective sleep quality • ↑ N1 sleep and normal N2 and N3 relative to typical levels (no control group) • ↑ SOL
Ingram	2023	Human: parent report	CSHQ	22q11.2DS = 100	2–17	• High rates of sleep problems • Sleep problems associated with daytime behavior problems
• Reich	2023	Human: actigraphy and parent-report	• Actigraphy • CSHQ	22q11.2DS = 69 TD = 38	5–34	• Non-restorative sleep pattern present in 22q11.2DS • Non-restorative sleep associated with affective and psychotic symptoms
Chawner	2023	Human: parent report	Tayside Children’s Sleep Questionnaire (TCSQ)	22q11.2DS = 32 TD = 12	2–6	• Sleep problems manifest at an early age in 22q11.2DS • Sleep problems associated with behavioral and emotional problems
Leader	2023	Human: parent report	The Sleep Questionnaire	22q11.2DS = 101	8–60	• High rates of sleep problems, daytime sleepiness, and snoring in 22q11.2DS • ↑Sleep problems correlated with ↑ psychosis, depression, and anxiety
• Maurer	2020	Drosophila: behavior	Locomotion during consistent light/dark cycles	Drosophila *LZTR1* knockdown	Adult	• Neuronal knockdown of *LZTR1* “night owl” gene in the 22q11.2 locus resulted in ↓ nighttime sleep • Knockdown in GABA neurons sufficient
Saito	2020	Mouse: behavior	Locomotion during changing light/dark cycles	*Del(3.0 Mb)/* + and WT mice	Adult	• Faster adaptation to experimental jet lag in *Del(3.0 Mb)/ + *compared to WT mice • ↓ Activity at night in Del(3.0 Mb)/ + mice
Saito	2021	Mouse: EEG	Intracranial sleep EEG/EMG	*Del(3.0 Mb)/* + and WT mice	Adult	• ↓ EEG theta power during REM sleep in Del(3.0 Mb)/ + mice • ↓ Wakefulness and ↑ non-REM sleep during the first few hours of night in Del(3.0 Mb)/ + mice

### Sleep-Disordered Breathing

Rates of SDB are increased in 22q11.2DS due to high rates of craniofacial and palatal abnormalities [30, 31], but the increase in risk is modest, with the prevalence rates ranging from 5–20% [21, 30, 32], compared to 1–3% in the general pediatric population [33, 34] and 14.5% in the general adult population [35]. However, there have been subjective reports of increased symptoms of SDB, such as snoring or sleepiness, in 22q11.2DS [21, 24]. The proportion of parents reporting SDB symptoms in their child on a subjective questionnaire was 49%; however, these reports were not confirmed with PSG [26]. One PSG study of 22q11.2DS reported an elevated apnea–hypopnea index (AHI) in 2 of 20 individuals assessed [27•]. SDBs such as obstructive sleep apnea (OSA) are an important treatment target for affected individuals with 22q11.2DS, but are not likely to be the primary source of sleep disturbances in 22q11.2DS [23•].

### Parasomnias

While little information exists on sleep disorder diagnoses in 22q11.2DS, a handful of studies report on parasomnias. Parasomnias are sleep disorders defined by undesirable physical events occurring during sleep initiation, sleep, or arousal from sleep [36]. One particular parasomnia of interest is rapid eye movement (REM) behavior disorder (RBD), which involves dream enactment due to the lack of paralysis during REM sleep. The presence of RBD can be an early marker of some neurodegenerative disorders [37, 38] such as Parkinson’s disease, which recent evidence suggests is more common in 22q11.2DS [39]. There are several reports of elevated scores on questionnaires measuring symptoms of RBD [27•] and general parasomnias [21, 24, 26] in individuals with 22q11.2DS compared to controls. However, in a sample of adults with 22q11.2DS, no individual met criteria for RBD when measuring REM sleep without a loss of muscle tone (atonia), an objective marker of RBD on a PSG [27•]. Further, there were no differences in subjectively reported parasomnias compared to sibling controls in one study [23•].

### Multivariate Patterns of Sleep Disruption

Two studies [23•, 29•] took a multivariate approach to identify sleep patterns associated with 22q11.2DS. The first [23•], using items from a clinician-rated sleep assessment based on parent report, identified two distinct patterns of sleep disruptions. A restless sleep pattern identified in 22q11.2DS included items related to hypersomnia, restless sleep, inadequate rest, tiredness, and fatiguability. An insomnia sleep pattern included items related to initial, middle, and early insomnia. Another study [29•] utilized items from the CSHQ and sleep continuity measured derived from actigraphy (WASO, SOL, SE, TIB, number of awakenings, average awakening length, in bed time, and out bed time) to identify a pattern of non-restorative sleep in 22q11.2DS, which was characterized by earlier in-bed time, longer TIB, reduced number of awakenings, longer TST, and higher subjectively reported daytime fatigue.

### Age and Sex Differences

While no studies have primarily focused on the effects of age and sex on sleep disturbances in 22q11.2DS, several studies report secondary analyses of age and sex effects on sleep. With regards to sex differences, one study [21] found that females reported more sleep anxiety and night waking than males, while another study [25•] found that males reported later bedtimes than females. With regards to age, parent reports indicated that younger subjects with 22q11.2DS had increased bedtime resistance, night wakings, and SDB, as well as decreased SOL, compared to older subjects [21]. In another study, younger subjects exhibited increased sleep duration and earlier waking in subjects with 22q11.2DS [25•].

## Correlates of Sleep Behaviors

Understanding sleep behavior disruptions is important because they are often correlated with mental and physical symptoms that impact people’s daily lives. In TD populations, sleep disruptions are associated with increased levels of anxiety [40, 41]; depression [41]; behavioral problems [42, 43]; chronic pain, inflammation, and immune dysfunction [44]; cognitive difficulties [45]; and an overall decreased quality of life [46]. As in other populations, sleep disturbances are closely related to psychiatric well-being and behavioral problems in 22q11.2DS [20, 23•, 24, 25•, 26].

### Psychiatric and Neurodevelopmental Disorders

In 22q11.2DS, subjective reports of poor sleep have been associated with diagnoses of neurodevelopmental disorders, such as ASD and ADHD [23•], and more severe dimensionally measured symptomology[20, 23•, 24], including restricted and repetitive behaviors [20, 24], and social behavior [20]. Similar to studies of idiopathic ASD, two studies reported that worse overall sleep in 22q11.2DS was associated with more severe self-injurious, stereotyped behaviors, and aggressive behavior [20, 24]. Moulding et al. [23•] found that a restless sleep pattern was associated with symptoms of ADHD and a conduct disorder, while an insomnia sleep pattern was associated with symptoms of conduct disorder. Similarly, a recent study found that worse sleep as assessed by the CSHQ was associated with increased behavioral problems and speech delay in 22q11.2DS [26].

The relationship between sleep and psychosis risk in 22q11.2DS is of particular interest because of the greatly increased risk of a PSD in 22q11.2DS and the close relationship between sleep disturbance and psychosis [47–50]. Consistent with literature on sleep disturbances in idiopathic schizophrenia and youth at clinical high-risk for psychosis (CHR) [48, 51, 52], increased psychosis risk symptoms were related to worse sleep disturbances in individuals with 22q11.2DS [20]. In a smaller sample, there were no differences in the severity of sleep disturbances reported between individuals with 22q11.2DS who met criteria for a PSD and those who did not, but those with PSD reported worse sleep-related daytime impairment on the PSQI than those without PSD [22•].

Hyde et al. [25•] investigated the causal relationship between sleep and affect in individuals with 22q11.2DS using daily sleep and affect measurements. Overall, the number of mental health diagnoses was associated with increased SOL. They found that sleep had a bi-directional relationship on affect such that poor sleep reported on a sleep diary predicted worse next-day affect, and negative affect was associated with worse quality of the coming night’s sleep. Interestingly, there were group differences in these relationships, such that the 22q11.2DS group exhibited stronger relationships than controls in sleep-based prediction of next-day irritation and negative affect. Similarly, there was a stronger positive relationship in 22q11.2DS between suspiciousness predicting coming night’s SOL as well as loneliness and irritation predicting the number of awakenings in coming night’s sleep.

### Cognition

Sleep has a well-documented relationship with cognition, particularly due to mechanisms of memory consolidation that occur during sleep [53, 54]. In individuals with 22q11.2DS, poor sleep has also been found to be associated with worse complex cognition, episodic memory, motor praxis [22•], sustained attention, and set-shifting performance [23•], as well as poorer real-world executive functioning [20]. However, based on findings to date, there is no reported association between sleep and full-scale IQ in 22q11.2DS [23•].

### Physical Health

In addition to mental health, subjectively reported sleep problems are also related to measures of physical health in individuals with 22q11.2DS [22•, 24]. In children and adolescents with 22q11.2DS, increased sleep problems were associated with worse self-reported gastrointestinal (GI) symptoms, such as bloating, nausea, and abdominal pain, which are common in individuals with 22q11.2DS [55]. However, due to the cross-sectional nature of this study, the causal direction of the relationship between sleep and GI symptoms is unclear. In the general population, consistent, reciprocal relationships have been demonstrated between sleep and the immune system, indexed by inflammatory cytokines. For example, sleep deprivation results in increased expression of inflammatory cytokines, and increased activity of inflammatory cytokines in the brain increases sleep duration [44, 56]. Yirimiya et al. [22•] examined the relationship between PSQI scores and levels of inflammatory cytokines in the blood, including interleukin 1ra, interleukin 6 (IL-6), interleukin 10 (IL-10), and tumor necrosis factor alpha. They found that increased levels of the inflammatory cytokine IL-6 were related to worse sleep problems in individuals with 22q11.2DS without PSD, but not those with PSD. There were no significant associations between sleep and the other cytokines tested. These studies demonstrate that sleep disturbances are linked to physical health, in addition to cognition and mental health; in 22q11.2DS however, more in-depth investigation is required to fully understand the complex relationship between sleep and immune dysfunction 22q11.2DS.

## Neurophysiological and Genetic Mechanisms

There is clear evidence that sleep behaviors are disrupted across the lifespan in individuals with 22q11.2DS, and these sleep disturbances are associated with the severity of multiple physical and mental health problems common in the disorder [19–21, 22•, 23•, 24, 25•, 26]. In particular, a common theme of disrupted or non-restorative sleep has emerged in the literature examining sleep behaviors in 22q11.2DS. Non-restorative sleep could result from sleep disorders (e.g., SDB or parasomnias), medical comorbidities (e.g., heart disease or arthritis), or dysregulation of neural circuits regulating sleep. Studies of behavioral sleep patterns suggest disrupted sleep physiology in 22q11.2DS, but cannot provide insight into the biological mechanisms contributing to the sleep disturbances. Therefore, study of sleep architecture and neurophysiology in 22q11.2DS using overnight EEG is required to determine brain mechanisms contributing to the observed sleep dysfunction. However, only two studies examining sleep architecture and one study of sleep neurophysiology exist to date in 22q11.2DS [27•, 28••].

### Sleep Architecture

The first study of sleep architecture in 22q11.2DS included 26 adults who completed an overnight clinical PSG in a sleep lab. Sleep architecture refers to the structural organization of sleep. Sleep is composed of non-rapid eye movement (NREM) and REM sleep. NREM sleep is further divided into stages N1 (“light” sleep), N2, and N3 (“deep” or “slow wave” sleep). Although there was no comparison group, they found that individuals with 22q11.2DS exhibited a higher proportion of N1 sleep and increased REM latency compared to typical levels [27•], which is consistent with prior reports on non-restorative sleep in 22q11.2DS. Contrastingly, a subsequent sleep EEG study [28••], which reported on 28 individuals with 22q11.2DS and 18 TD sibling controls who completed an overnight sleep assessment with full-scalp EEG in their own homes, reported that individuals with 22q11.2DS spent less time in N1 sleep and more time in stage N3 and REM than controls. Increased total power in fronto-lateral regions during stages N2, N3, and REM was also observed in 22q11.2DS compared to controls.

### Sleep Neurophysiology

**Fig. 1:**
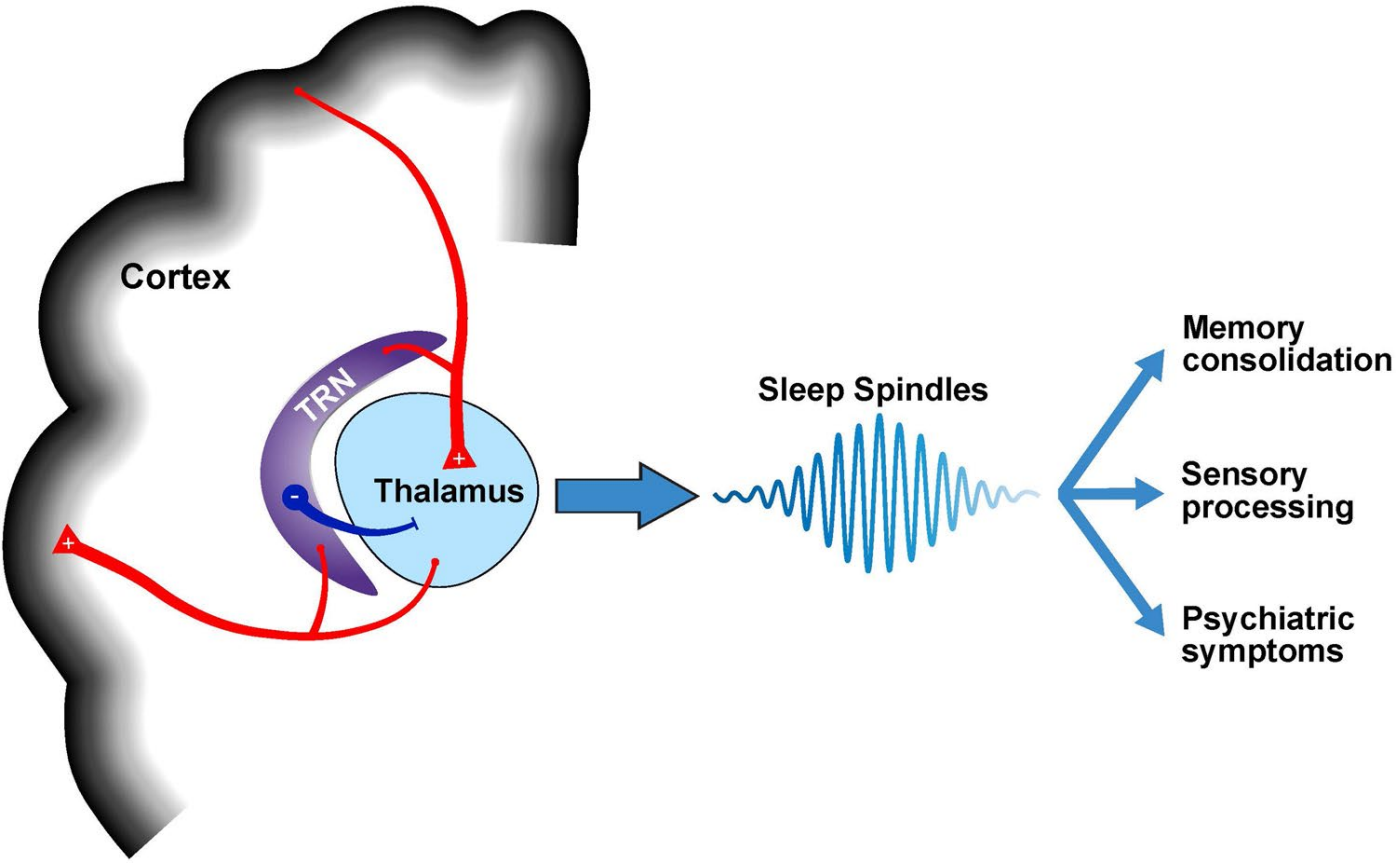
Schematic of thalamocortical circuitry underlying sleep spindle generation. Sleep spindles are generated in the thalamic reticular nucleus (TRN). Sleep spindles measured on the scalp through EEG arise from the inhibition of thalamocortical projections by the TRN (shown in blue), which generate reciprocal thalamocortical interactions (shown in red). These reciprocal thalamocortical interactions, measured by sleep spindles, play an important role in memory consolidation, sensory processing, and psychiatric symptoms

Sleep neurophysiology refers to brain functioning during sleep measured by EEG. Two important measures of sleep neurophysiology are slow waves (SW) and sleep spindles (Fig. 1). SWs are low-frequency oscillations that occur in the delta spectral band (0.4–4 Hz) during stage N3 sleep and reflect homeostatic sleep regulation by cortico-thalamic loops [54]. Sleep spindles are high-frequency bursts in the sigma band (11–16 Hz) in stages N2 and N3 that reflect activity in thalamocortical circuits, which are particularly important for sensory gating and sleep-dependent memory consolidation [53, 57, 58]. Examination of SWs, spindles, and their temporal relationship can provide valuable information about brain circuitry, which can in turn predict waking brain functions [59]. Particularly relevant to 22q11.2DS, there is a robust literature demonstrating that deficit in spindles (decreased density, duration, and amplitude) is a stable marker of schizophrenia diagnosis [59–61] that is associated with positive symptom severity [52, 62], decreased cognition (IQ, memory performance [52], sleep-dependent memory consolidation [63]), and resting-state connectivity between the thalamus and cortical sensory regions [59]. Therefore, studies of sleep neurophysiology in 22q11.2DS can both reveal biological insights into the physiology of sleep disturbances and their close relationship with clinical outcomes, as well as provide a genetic model of sleep physiology and psychosis risk which may be relevant to other NPDs.

In the only published study to date of sleep neurophysiology of 22q11.2 DS compared to TD controls to date, decreases in SW amplitude were observed in central, frontal, and lateral regions [28••]. There were no differences in delta power or SW density or duration reported. While the literature on SWs in other disorders is mixed, these findings are consistent with previous reports of SW deficits in children with idiopathic ASD [64, 65] and individuals with schizophrenia [66, 67]. However, several studies report no differences in SW characteristics in ASD [68] and schizophrenia [69, 70] populations compared to controls. Decreased sigma power has been observed in central regions during N2 and N3 sleep in 22q11.2DS [28••]; yet, contrary to findings in a CHR population [71], 22q11.2DS exhibited increases in three sleep spindle metrics: increased amplitude in fronto-lateral regions, density in central regions, and frequency in central and occipital regions. Further, there was a more consistent spindle-SW phase relationship observed in 22q11.2DS compared to TD controls. Interestingly, the differences in SW-spindle coupling observed in 22q11.2DS are inconsistent with reports in CHR [71], but similar to those found in idiopathic ASD [68].

In control subjects, increased amplitude of both SWs and sleep spindles are predictive of increased sleep-dependent memory consolidation, measured as greater overnight improvement on a spatial cognition task [28••]. However, individuals with 22q11.2DS failed to show these associations, despite exhibiting higher spindle and SW amplitudes than the control group and showing similar overnight improvements in spatial cognition task performance. A similar phenomenon was reported in a recent study of idiopathic ASD [68]: increased sleep spindle density was associated with less overnight improvement on a spatial memory task in individuals with ASD, but the opposite relationship was found in the TD group, despite both groups showing overnight improvement in task performance. Interestingly, in the 22q11.2DS study, mediation analyses revealed that sleep spindle amplitude mediated the effect of CNV status on ASD and both SW amplitude and consistency of spindle-SW coupling mediated the effect of CNV status on both ADHD and anxiety [28••]. While further research is required to fully understand the mechanisms of the relationship between the 22q11.2 deletion, sleep neurophysiology, and developmental neuropsychiatric disorders, this finding suggests that neurobiological functions performed during sleep serve an important role in NPDs.

### Thalamocortical Circuits

SWs and sleep spindles reflect reciprocal interactions in thalamocortical feedback loops (Fig. 1; see [54]). Therefore, the observed differences in sleep neurophysiology in 22q11.2DS compared to TD individuals likely reflect thalamocortical dysfunction, which has been reported in resting-state functional magnetic resonance imaging (rs-fMRI) studies of 22q11.2DS [72, 73] as well as idiopathic ASD [74–77] and schizophrenia [78]. However, the direction of the effect found in 22q11.2DS is perplexing; a sleep spindle deficit, particularly decreased spindle density, has been shown to be associated with hyperconnectivity between the thalamus and somatomotor regions in the cortex in schizophrenia [59]. Yet, in the only published study to date, individuals with 22q11.2DS exhibited *increased* sleep spindle amplitude, density, and frequency, despite previous reports of hyperconnectivity between the thalamus and cortical somatomotor network in that population [72]. A recent longitudinal rs-fMRI investigation of thalamocortical connectivity in 22q11.2DS found a steady decline of connectivity between the thalamus and cortical somatomotor network from ages 6 to 23, such that younger individuals exhibited hyperconnectivity compared to TD controls, while older individuals exhibited hypoconnectivity compared to TD controls [73]. Therefore, the effect of age on thalamocortical connectivity in 22q11.2DS, which is consistent with the increase in sleep spindle density observed in older individuals with 22q11.2DS [28••], may explain this discrepancy. It also may be possible that the neural correlates of sleep spindles differ in 22q11.2DS compared to other populations, as no study to date has investigated the relationship between sleep spindles and rs-fMRI measures of thalamocortical connectivity in 22q11.2DS. This notion is supported by the absence of a relationship between sleep spindles and sleep-dependent memory consolidation in 22q11.2DS [28••]. There are several studies spanning species [53, 79] and clinical populations demonstrating the beneficial role of sleep spindles in sleep-dependent memory consolidation [69, 80], so the absence of this relationship in both 22q11.2DS [28••] and ASD [68] suggests that perhaps the thalamic mechanisms underlying sleep spindles may have different rs-fMRI and cognitive correlates in 22q11.2DS compared to other populations. Future research should concurrently investigate sleep spindles, thalamic connectivity, and cognition in 22q11.2DS to better understand their interactions.

### Pre-clinical Models

Another method of probing mechanisms underlying sleep disturbances in 22q11.2DS is through pre-clinical genetic models. A study in *drosophila* [81•] found a gene in the 22q11.2 locus that is required for nighttime sleep initiation and maintenance, LZTR1 (night owl; *nowl*). Knockdown of this gene resulted in delayed sleep initiation and increased sleep fragmentation. However, two studies in mouse models of 22q11.2DS [82, 83] found differences in circadian behaviors. Additionally, 22q11.2DS model mice were found to have increased NREM sleep during the first few hours of dark periods and decreased theta power during REM sleep relative to wild-type mice. It is difficult to draw conclusions about the genetic influence of a deletion in the 22q11.2 locus on sleep disturbances given the available data, but these preliminary studies of animal models suggest that sleep disturbances in 22q11.2DS may be attributable in part to conserved genetic mechanisms.

## Sleep as a Treatment Target

Sleep represents an exciting potential intervention target because of its association with many different mental and physical health symptoms, across domains, that are frequently reported in 22q11.2DS. It is an exciting possibility that a single sleep intervention could target several troublesome symptoms and improve overall quality of life for individuals with 22q11.2DS and their families. However, there is very little data available to date on sleep interventions in 22q11.2DS, so it is unclear to what degree sleep interventions can be effectively applied in this population. Determining the efficacy of sleep interventions in 22q11.2DS is an important future direction due to growing evidence in other populations with psychiatric and medical comorbidities that behavioral sleep interventions, such as cognitive behavioral therapy for insomnia (CBT-I), can not only improve sleep [12, 84–88], psychiatric symptoms [12, 84–88], physical health conditions [87, 88], and overall quality of life [84], but also prevent worsening of psychiatric symptoms [89]. This suggests that sleep interventions may be a beneficial early treatment for subjects with 22q11.2DS, but more research is required.

A handful of studies with data on sleep medication report high use of pharmacological sleep aids in the 22q11.2DS population [23•]. However, pharmacological sleep aids have the potential for abuse, withdrawal, and harmful side effects [90]. Therefore, improved interventions targeting specific aspects of sleep are required.

While the field is still nascent, certain aspects of sleep neurophysiology, like sleep spindles and SWs, have been shown to be modifiable through pharmacological (e.g., zolpidem [91]) and non-pharmacological methods (e.g., closed-loop auditory stimulation [92]). These methods represent a potential intervention targeting specific brain mechanisms involved in non-restorative sleep, like abnormalities in SW or sleep spindles. However, these methods of manipulating sleep have not yet been tested in 22q11.2DS. Future research is required to test the efficacy of well-validated sleep interventions, such as CBT-I, in 22q11.2DS and develop new approaches to target specific aspects of sleep neurophysiology that are altered in 22q11.2DS.

## Challenges and Future Directions for the Field

A consistent pattern in the sleep literature in 22q11.2DS is the discrepancy between results obtained from subjective and objective sleep measures. While this discrepancy is fairly common, particularly in clinical populations, several potential causes should be considered in studies of 22q11.2DS. First, it is unclear whether subjective sleep measures validated in normative populations apply to individuals with 22q11.2DS, who often exhibit DD and ID. Further, many studies employ a single questionnaire with a single administration method in samples with wide age ranges, but it is unclear whether self or parent report is appropriate due to the high heterogeneity within 22q11.2DS. For example, a young adult with 22q11.2DS and minimal cognitive impairment may live independently while a young adult with ID may live with their parent. A parent-report questionnaire may be appropriate for the young adult with ID, but not for the individual who is independently living. Mauro et al. [27•] found no correlations between PSG measures and PSQI scores. But due to the scarcity of reports of objective measures of sleep in this population, it is unclear whether this discrepancy results from different aspects of sleep being captured in subjective versus objective measures, or if it is suggestive of low validity of standardized sleep questionnaires in 22q11.2DS. Future research in this population should prioritize sleep measures which could be administered as self or parent report depending on the individual, not based solely on age ranges suggested by the instrument. Further, a comprehensive study of sleep in 22q11.2DS utilizing multimodal sleep measures is required to gain holistic insight into sleep in 22q11.2DS.

The primary challenge to address in the field of sleep in 22q11.2DS, and clinical sleep research more broadly, is the high patient burden and logistical difficulty of gold standard methods of measuring sleep. PSG often takes place in an unfamiliar environment and requires individuals to sleep with several sensors attached to their face, scalp, and body. However, due to the elevated rates of sensory processing problems and anxiety in 22q11.2DS, the gold standard PSG methods are nearly impossible for many individuals. Those with 22q11.2DS who are able to tolerate PSG thus represent a limited subset of the broader spectrum of symptoms and clinical presentations encountered in the syndrome. In TD populations, PSG methods require an adaptation night to ensure there is no effect of the PSG equipment on sleep. Populations with NDDs have been shown to require additional adaptation nights [93]. The two studies employing PSG in 22q11.2DS did not include an adaptation night, and report on small samples, which likely contributes to the discrepancies in the literature outlined above. To advance the field of sleep research in 22q11.2DS and to harness the great potential of sleep neurophysiology as a scalable biomarker, a low-burden measure of sleep neurophysiology is required.

Promising future directions for sleep research in 22q11.2DS include larger studies with longitudinal follow-up that integrate actigraphy, targeted measures of sleep behavior, non-invasive technologies for measuring sleep architecture and neurophysiology, and other neuroimaging modalities. Clinical trials of potential interventions for disrupted sleep in 22q11.2DS are also a priority. Clinical advances will hopefully also be facilitated by future pre-clinical research investigating sleep phenotypes, underlying neurobiology, and potential interventions in in vitro and animal models of 22q11.2DS.

## Conclusion

There is convergent evidence that sleep is widely disrupted in 22q11.2DS, and sleep disruptions are associated with several aspects of psychiatric, behavioral, and physical well-being. However, the literature to date is sparse and primarily relies on subjectively reported measures of sleep. It remains unclear whether the sleep disruptions in 22q11.2DS are a core feature of the genetic disorder, or are better accounted for by the NDDs and psychiatric disorders present in the population. Study of sleep neurophysiology suggests that understanding the mechanisms of brain activity during sleep reveals rich information about underlying neurobiology and risk for psychiatric disorders. Studying sleep in 22q11.2DS thus represents an important opportunity for clinically impactful discovery.
